# Application of a hybrid wavelet feature selection method in the design of a self-paced brain interface system

**DOI:** 10.1186/1743-0003-4-11

**Published:** 2007-04-30

**Authors:** Mehrdad Fatourechi, Gary E Birch, Rabab K Ward

**Affiliations:** 1 Department of Electrical and Computer Engineering, University of British Columbia, Vancouver, BC V6T 1Z4, Canada; 2 Neil Squire Society, Burnaby, BC V5M 3Z3, Canada; 3 Institute for Computing, Information and Cognitive Systems, Vancouver, BC V6T 1Z4, Canada

## Abstract

**Background:**

Recently, successful applications of the discrete wavelet transform have been reported in brain interface (BI) systems with one or two EEG channels. For a multi-channel BI system, however, the high dimensionality of the generated wavelet features space poses a challenging problem.

**Methods:**

In this paper, a feature selection method that effectively reduces the dimensionality of the feature space of a multi-channel, self-paced BI system is proposed. The proposed method uses a two-stage feature selection scheme to select the most suitable movement-related potential features from the feature space. The first stage employs mutual information to filter out the least discriminant features, resulting in a reduced feature space. Then a genetic algorithm is applied to the reduced feature space to further reduce its dimensionality and select the best set of features.

**Results:**

An offline analysis of the EEG signals (18 bipolar EEG channels) of four able-bodied subjects showed that the proposed method acquires low false positive rates at a reasonably high true positive rate. The results also show that features selected from different channels varied considerably from one subject to another.

**Conclusion:**

The proposed hybrid method effectively reduces the high dimensionality of the feature space. The variability in features among subjects indicates that a user-customized BI system needs to be developed for individual users.

## Background

A successful brain interface (BI) system enables individuals with severe motor disabilities to control objects in their environment (such as a light switch, neural prosthesis or computer) by using only their brain signals. Such a system measures specific features of a person's brain signal that relate to his or her intent to affect control, then translates them into control signals that are used to control a device [1,2].

Brain interface systems are implemented in two ways: system-paced (synchronized) or self-paced (asynchronous). In system-paced BI systems, a user can initiate a command only during certain periods specified by the system. In a self-paced BI system, users can affect the output of the BI system whenever they want, by intentionally changing their brain state. The state in which a user is intentionally attempting to control a device is called an *intentional control *(IC) state. At other times, users are said to be in a *no-control *(NC) state, where they may be idle, thinking about a problem, or performing some action other than trying to control the device[3,4]. To operate in this paradigm, BI systems should be designed to respond only when the user is in an IC state and to remain inactive when the user is in an NC state. So far, only a few BI systems (e.g. [3,5-10]) have been specifically designed and tested for self-paced control applications. But as recognized in [2], self-paced BI systems deserve more attention.

The discrete wavelet transform (DWT) can be used as a powerful feature extraction tool to extract time-frequency features similar in shape to that of a particular wavelet function. It therefore has an advantage over other feature extraction methods that operate in only one domain, such as the Fourier transform, or autoregressive modeling.

The DWT has been extensively applied in the analysis of event-related potential (ERP) because of its ability to effectively explore both the time and frequency information of these signals [11,12]. It has also been successfully used to generate wavelet features in BI systems. In [13], DWT was employed in the design of a system-paced BI system that used wavelet coefficients extracted from slow cortical potentials (SCPs) as well as other ERPs. This system performed better than other designs that used EEG time series and a mixed filtering method. In [14], the energies of various frequency bands decomposed by a wavelet packet transform (18 frequency bands in total) were used as features in detecting different movement patterns in a self-paced BI system. These features were linearly combined to generate a single feature, with coefficients of the linear mapping determined by a genetic algorithm (GA). In [15], a custom-made wavelet function was employed in two different studies: the detection of P300 in a single EEG channel, and the detection of the Bereitschafts potential from two EEG channels. In [16], a weighted linear combination of all available wavelet coefficients (15 in total) extracted from a single EEG channel was used to detect P300 patterns. To estimate weights for each feature in the linear combination, a neural network was employed. Finally, in [17], DWT was applied to extract the 0–4 Hz component of the EEG signal in a P300-based BI system. Based on the above encouraging results, in this study we explore applying DWT to extract movement-related potential (MRP) features for driving a self-paced BI system.

Although the above BI studies provide promising evidence that DWT can be employed to extract features in BI systems, two main issues still need to be addressed. First, studies that used discrete wavelet coefficients as features (rather than *wavelet-filtered EEG signals*), used only one or two EEG channels. In these cases, the resulting dimensionality of the space does not pose a serious problem, since it is not very large. Having a BI system that uses data recorded from only one or two electrodes seems very appealing, since the setup is fast and uses less hardware/software infrastructure. Most of the above-mentioned papers, however, achieved a relatively high degree of classification error when only one or two EEG channels were used. For example, in [16], the reported error rates were relatively high (nearly 40% error). In [17], where wavelet-filtered EEG signals were used, the system did not perform well (30% misclassification). For the only self-paced BI system that has applied wavelet coefficients so far [14], false activation rates (the percentage of hits that were not true positives) varied up to 67%, however, the authors did not indicate the number of NC epochs used in their study, so critical commentary on the performance of their BI system cannot be made. The invasiveness of the recording technology of the BI system in [14] is also an important issue that needs to be considered.

The above observations strongly motivate the use of additional EEG electrodes in BI systems. With signals recorded from multiple channels, we can explore spatial information, which is expected to yield improvements in classification performance.

Another issue that must be addressed when using DWT to extract features in BI systems is the feature selection procedure. That is, how many features should be selected and how should they be selected? In [13], all of the 64 wavelet features used for classification were extracted from only one EEG channel. In [15], because of the computational limitations affecting the classifier, only a number of top wavelet features (ranked by the amount of discriminability) were selected. None of the above-mentioned approaches yielded best results (since the feature selection process used was not necessarily optimal). Using all features does not necessarily provide the best results, because some of the less discriminant features may degrade the classifier's performance [18]. On the other hand, using only few features that have the highest rank (and filtering out the rest of features) does not necessarily lead to optimal classification performance, since there is no guarantee that using only top-ranked features leads to the best classifier performance[19].

Based on the related literature review, we postulate that the information extracted from *multiple-electrode signals *is necessary for achieving acceptable performance. This in turn leads us to the high dimensionality problem of the feature space, since the feature space dimension is directly affected by the number of electrodes used as well as by the number of features per EEG signal. Since not all the wavelet coefficients provide discriminatory information between the output classes, we postulate that features that better discriminate between the output classes need to be selected to obtain better classification performance. A mechanism for selecting the most discriminating features is thus needed.

Wrapper methods, such as GAs, use the classifier's performance to evaluate a particular feature vector. They provide a good solution for finding the features that work well together by choosing the ones that lead to better classifier performance [20]. The downside of using wrapper methods is time inefficiency. As the dimension of the search space increases, it becomes harder for a wrapper method to find a suitable subset of features that lead to a high performance.

In order to benefit from the advantages of both filter and wrapper methods, we decided to employ a hybrid approach. Features carrying the least discriminative information about the output classes were filtered out first. Then a wrapper method was applied to the reduced feature space to find the features that work well together, i.e., the combination that leads to the best classification performance. We used mutual information (MI) in the filtering stage. Mutual information is a powerful tool for ranking features based on the amount of discriminative information each carries [21]. We then applied a GA in a wrapper approach to select the features that lead to the best classification performance. Genetic algorithms are heuristic methods that can effectively sample large search spaces [22]. They are implemented based on the principles of evolutionary biology, and evolve over many generations. By mimicking this process, GAs are able to evolve solutions to real-world problems. They have been shown to be useful tools in automatically customizing many practical systems [22,23].

We used a support vector machine (SVM) to classify the selected features into one of two classes: no control (NC) or intentional control (IC). The results of this study show that applying the proposed approach to the offline data collected from four able-bodied subjects yields low false positive (FP) rates at a reasonably high true positive (TP) rate. We also examine the spatial distribution of the selected features. We show that this distribution varies considerably from one subject to another. This finding shows the importance of user customization of BI systems.

### Data collection

People with severe motor disabilities cannot physically execute certain movements such as a finger flexion, but they are usually able to *attempt *it. Several studies have shown that recordings of brain signals obtained from attempted and real movements for able-bodied subjects bear many similarities [14,24-29]. Based on these studies, both attempted and executed movements have been shown to activate similar cortical areas and to generate similar movement patterns. This evidence enables us to base our analysis on the data of able-bodied subjects, who actually execute the particular movement. It is then possible to detect the occurrence of the control command by analyzing signals such as electro-myographic (EMG) signal or the output of an actual switch. Such signals can be used to label the brain signals and to evaluate the performance of a BI. The data analysis of individuals with motor disabilities was thus left to future studies.

The data of four (three male and one female) able-bodied subjects were used in this study. All subjects were right-handed and between 31 and 56 years old. They had all signed consent forms prior to participation in the experiment.

Subjects were positioned 150 cm in front of a computer monitor. The EEG signals were recorded from 13 monopolar electrodes positioned over the subjects' supplementary motor area and primary motor cortex (according to the International 10–20 System at F_1_, F_z_, F_2_, FC_3_, FC_1_, FC_z_, FC_2_, FC_4_, C_3_, C_1_, C_z_, C_2 _and C_4 _locations). Electro-oculographic (EOG) activity was measured as the potential difference between two electrodes, placed at the corner of and below the right eye. An ocular artifact was considered present when the difference between the EOG electrodes exceeded ± 25 μV. All signals were sampled at 128 Hz and referenced to ear electrodes (see [30] for details of the data recording). The recorded signals were then saved on the computer and converted to bipolar EEG signals by calculating the difference between the adjacent EEG channels. This procedure was used since it has been shown that bipolar electrodes generate more discriminating features than do monopolar electrodes [3]. This conversion generated the following 18 bipolar EEG channels: F_1_-FC_1_, F_1_-F_z_, F_2_-F_z_, F_2_-FC_2_, FC_3_-FC_1_, FC_3_-C_3_, FC_1_-FC_z_, FC_1_-C_1_, FC_z_-FC_2_, C_1_-C_z_, C_2_-C_4_, FC_2_-FC_4_, FC_4_-C_4_, FC_2_-C_2_, FC_z_-C_z_, C_3_-C_1_, C_z_-C_2 _and F_z_-FC_z_.

The data were collected from subjects as they performed the following guided task. At each interval, a white, 2 cm diameter circle was displayed on the subject's monitor for 1/4 second, prompting the subject to attempt a movement. In response to this cue, the subject had to perform a right index finger flexion one second after the cue appeared. The 1-second delay was used to avoid visual evoked potential (VEP) effects caused by the cue (see [31] for more details). For each subject, 80 IC epochs were collected on average every day over a period of 5 days.

An IC epoch consisted of data collected over an interval containing the movement onset (measured as the finger switch activation) if no artifact was detected in that particular interval. The interval starts at *t*_*start *_seconds before movement onset and ends at *t*_*finish *_seconds after it. There were limitations in choosing the total length of (*t*_*start *_+ *t*_*finish*_). If the length of (*t*_*start *_+ *t*_*finish*_) increases, more artifacts may be present in an IC epoch. As a result, the number of training epochs that are artifact-free based on the criterion used to reject ocular artifacts will be reduced. If the length of (*t*_*start *_+ *t*_*finish*_) is too short, a poor exploration of potential features results. Since a simple finger flexion MRP usually starts about 1.5 seconds before the movement and returns back to the normal baseline around 1 second after the movement [32], the data obtained from 1.5 seconds before to 1.0 second after the movement onset were analyzed (i.e., *t*_*start *_= 1.5 seconds and *t*_*finish *_= 1.0 second).

The NC epochs were selected as follows. A window of width (*t*_*start *_+ *t*_*finish*_) seconds was considered (*t*_*start *_= 1.5 seconds and *t*_*finish *_= 1.0 second). To extract NC epochs, the window was shifted over each EEG signal recorded during NC sessions by a step of 16 samples (0.1250 sec). Wavelet coefficients were extracted for each epoch that did not contain artifacts.

## Method

**Figure 1:**
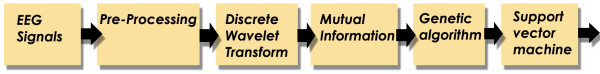
The overall structure of the proposed method.

The overall structure of the proposed scheme is shown in Figure 1. EEG signals were checked for the presence of EOG artifacts. The contaminated epochs were rejected, as explained in the "Data Collection" Section.

The continuous wavelet transform (CWT) is defined as the convolution of the signal *x*(*t*) with the wavelet functions *ψ*_*a,b*_(*t*) where *ψ*_*a,b*_(*t*) is the dilated and shifted version of the *wavelet function ψ*(*t*) and is defined as follows:

ψa,b(t)=a⋅ψ(t−ba)
 MathType@MTEF@5@5@+=feaafiart1ev1aaatCvAUfKttLearuWrP9MDH5MBPbIqV92AaeXatLxBI9gBaebbnrfifHhDYfgasaacH8akY=wiFfYdH8Gipec8Eeeu0xXdbba9frFj0=OqFfea0dXdd9vqai=hGuQ8kuc9pgc9s8qqaq=dirpe0xb9q8qiLsFr0=vr0=vr0dc8meaabaqaciaacaGaaeqabaqabeGadaaakeaaiiGacqWFipqEdaWgaaWcbaGaemyyaeMaeiilaWIaemOyaigabeaakiabcIcaOiabdsha0jabcMcaPiabg2da9maakaaabaGaemyyaegaleqaaOGaeyyXICTae8hYdKNaeiikaGYaaSaaaeaacqWG0baDcqGHsislcqWGIbGyaeaacqWGHbqyaaGaeiykaKcaaa@4293@

where *a *and *b *are the scale and translation parameters, respectively. The CWT maps a signal of one independent variable *t *into a function of two independent variables *a*, *b*. This procedure is redundant and not efficient for algorithmic implementations. Therefore, it is more practical to define the wavelet transform at a discrete scale *a *and a discrete time *b *by choosing the set of parameters (this transform is called a discrete wavelet transform, or DWT), such that

*a*_*j *_= 2^-*j*^, *b*_*j,k *_= 2^-*j*^·*k *(*j*, *k *are integers)

The contracted versions of the wavelet function will match the high-frequency components of the original signal and the dilated versions will match the low-frequency oscillations. Then by correlating the original signal with the wavelet functions of different sizes, the details of the signal at different scales are obtained. The resulting correlation features can be arranged in a hierarchical scheme called multi-resolution decomposition [33]. Multi-resolution decomposition separates the signal into "details" at different frequency bands and a coarser representation of the signal called an "approximation".

For our study, the *rbio3.3 *wavelet from the B-spline family was chosen as the wavelet function because it has some similarities with the shape of the classic bipolar MRP pattern. Using a 5-level decomposition method resulted in wavelet coefficients corresponding to the following frequency bands (the sampling frequency was 128 Hz): [32-64], [16-32], [8-16], [4-8], [2-4], and [0–2] Hz.

Based on the previous findings in [3], which showed that MRP features are mostly located in the frequency range below 4 Hz, only the lowest frequency bands (i.e., 0–2 Hz and 2–4 Hz) were considered for further analysis of MRPs. Even with this reduced feature space, the resulting feature space dimension (*N*_*features*_), which is the product of the number of electrodes (*N*_*electrodes*_) and the number of wavelet features per EEG signal (*N*_*wavelet*_). That is, *N*_*features *_= *N*_*electrodes *_× *N*_*wavelet *_remained very high. Thus, a feature selection procedure had to be used that could select thefeatures that lead to optimal classification performance. This procedure should specify the selected EEG channels as well as the features selected per channel.

We devised a hybrid feature selection algorithm to meet these requirements. Mutual Information (MI) was employed in the filtering stage and a GA was then used to select the optimal set of features.

Although MI has been used elsewhere to filter out the less informative features [21,34], it is *not *usually successful at finding features that lead to optimal classification performance. This is because when there are more than three feature dimensions, the calculation of MI is computationally demanding, and impossible for large feature spaces (since the calculation of MI requires the joint probability of features in a high dimension) [21,34]. Thus, MI was only used in our algorithm to discard the least informative features based on the amount of information that each feature carries regarding the output classes.

The MI between the input feature vector **X **and the output classes **Y **was calculated as follows:

*I*(**X**, **Y**) = *H*(**Y**) - *H*(**Y**|**X**)

Where

H(Y)=−∑j=1MP(yj)⋅log⁡2P(yj)
 MathType@MTEF@5@5@+=feaafiart1ev1aaatCvAUfKttLearuWrP9MDH5MBPbIqV92AaeXatLxBI9gBaebbnrfifHhDYfgasaacH8akY=wiFfYdH8Gipec8Eeeu0xXdbba9frFj0=OqFfea0dXdd9vqai=hGuQ8kuc9pgc9s8qqaq=dirpe0xb9q8qiLsFr0=vr0=vr0dc8meaabaqaciaacaGaaeqabaqabeGadaaakeaacqWGibascqGGOaakcqqGzbqwcqGGPaqkcqGH9aqpcqGHsisldaaeWbqaaiabdcfaqjabcIcaOiabdMha5naaBaaaleaacqWGQbGAaeqaaOGaeiykaKcaleaacqWGQbGAcqGH9aqpcqaIXaqmaeaacqWGnbqta0GaeyyeIuoakiabgwSixlGbcYgaSjabc+gaVjabcEgaNnaaBaaaleaacqaIYaGmaeqaaOGaemiuaaLaeiikaGIaemyEaK3aaSbaaSqaaiabdQgaQbqabaGccqGGPaqkaaa@4CC7@

H(Y|X)=−∑i=1N∑j=1MP(xi)⋅P(yj|xi)⋅log⁡2P(yj|xi)
 MathType@MTEF@5@5@+=feaafiart1ev1aaatCvAUfKttLearuWrP9MDH5MBPbIqV92AaeXatLxBI9gBaebbnrfifHhDYfgasaacH8akY=wiFfYdH8Gipec8Eeeu0xXdbba9frFj0=OqFfea0dXdd9vqai=hGuQ8kuc9pgc9s8qqaq=dirpe0xb9q8qiLsFr0=vr0=vr0dc8meaabaqaciaacaGaaeqabaqabeGadaaakeaacqWGibascqGGOaakieqacqWFzbqwcqGG8baFcqWFybawcqGGPaqkcqGH9aqpcqGHsisldaaeWbqaamaaqahabaGaemiuaaLaeiikaGIaemiEaG3aaSbaaSqaaiabdMgaPbqabaGccqGGPaqkcqGHflY1cqWGqbaucqGGOaakcqWG5bqEdaWgaaWcbaGaemOAaOgabeaakiabcYha8jabdIha4naaBaaaleaacqWGPbqAaeqaaOGaeiykaKIaeyyXICTagiiBaWMaei4Ba8Maei4zaC2aaSbaaSqaaiabikdaYaqabaGccqWGqbaucqGGOaakcqWG5bqEdaWgaaWcbaGaemOAaOgabeaakiabcYha8jabdIha4naaBaaaleaacqWGPbqAaeqaaOGaeiykaKcaleaacqWGQbGAcqGH9aqpcqaIXaqmaeaacqWGnbqta0GaeyyeIuoaaSqaaiabdMgaPjabg2da9iabigdaXaqaaiabd6eaobqdcqGHris5aaaa@6775@

P(yj)=∑i=1NP(xi)⋅P(yj|xi)
 MathType@MTEF@5@5@+=feaafiart1ev1aaatCvAUfKttLearuWrP9MDH5MBPbIqV92AaeXatLxBI9gBaebbnrfifHhDYfgasaacH8akY=wiFfYdH8Gipec8Eeeu0xXdbba9frFj0=OqFfea0dXdd9vqai=hGuQ8kuc9pgc9s8qqaq=dirpe0xb9q8qiLsFr0=vr0=vr0dc8meaabaqaciaacaGaaeqabaqabeGadaaakeaacqWGqbaucqGGOaakcqWG5bqEdaWgaaWcbaGaemOAaOgabeaakiabcMcaPiabg2da9maaqahabaGaemiuaaLaeiikaGIaemiEaG3aaSbaaSqaaiabdMgaPbqabaGccqGGPaqkcqGHflY1cqWGqbaucqGGOaakcqWG5bqEdaWgaaWcbaGaemOAaOgabeaakiabcYha8jabdIha4naaBaaaleaacqWGPbqAaeqaaOGaeiykaKcaleaacqWGPbqAcqGH9aqpcqaIXaqmaeaacqWGobGta0GaeyyeIuoaaaa@4CF5@

In these formulae, *I *represents the mutual information between **X **and **Y**, where **X **= {*x*_*i*_}, (*i *= 1,2,3,..., *N*) and **Y **= {*y*_*j*_}, (*j *= 1,2,3,..., *M*), *N *is the number of input states and *M *is the number of outputs states (*M *= *N *= 2, since the input and output can only take two values: IC and NC), *P*(*x*_*i*_) is the probability of occurrence of an input state *x*_*i*_, *P*(*y*_*j*_) is the probability of the output class *y*_*j *_when the input is unknown, and *P*(*y*_*j*_|*x*_*i*_) is the probability of the output class *y*_*j *_when the input state *x*_*i *_is known.

For each subject, the wavelet coefficient (feature) values corresponding to all the training set data were calculated. Then, using histograms with 10 bins each, the probability function of each feature was estimated and its mutual information with each of the output classes was calculated. The values of MI were calculated for all *N*_*features *_features and then ranked in descending order. The top *L *features were then selected. In this study, we arbitrarily chose *L *= 50 to avoid having a feature space with a very high dimension.

After reducing the dimension of the feature space, a GA was used to select a subset of *m *features from the top *L *features. To represent each possible combination of features, a binary chromosome of length *L *was defined. The bit *i *of the binary chromosome specified whether or not the feature *i *was selected by the GA. A value of "1" indicated the presence of feature *i *and a value of "0" indicated its absence in a chromosome.

An important decision in the design of a GA is the definition of a proper fitness function. In the proposed design, a suitable fitness function should consider at least three objectives: maximizing the TP rate, minimizing the FP rate and minimizing the number of features selected by the hybrid feature selection procedure.

The classification performance of a 2-state, self-paced BI system is usually determined by a confusion matrix, as shown in Table 1. In Table 1, the FP rate is the percentage of instances for which an NC epoch is misclassified as an IC epoch, the true negative (TN) rate is the percentage of NC epochs being correctly classified, the true positive (TP) rate is the percentage of IC epochs being correctly classified and the false negative (FN) rate is the percentage of misclassifying an IC epoch as an NC epoch. The fitness function should summarize this confusion matrix. For a 2-state self-paced BI system, we have

**Table 1:** The confusion matrix for a 2-state self-paced BI system.

**Actual Class/Predicted Class**	**IC**	**NC**
**IC**	TP	FN
**NC**	FP	TN

*FN*(%) = 100(%) - *TP*(%)

and

*TN*(%) = 100(%) - *FP*(%)

Based on equations (7) and (8), only TP and FP rates need to be included in the fitness function. One example of a fitness function is a function that maximizes the TPFP
 MathType@MTEF@5@5@+=feaafiart1ev1aaatCvAUfKttLearuWrP9MDH5MBPbIqV92AaeXatLxBI9gBaebbnrfifHhDYfgasaacH8akY=wiFfYdH8Gipec8Eeeu0xXdbba9frFj0=OqFfea0dXdd9vqai=hGuQ8kuc9pgc9s8qqaq=dirpe0xb9q8qiLsFr0=vr0=vr0dc8meaabaqaciaacaGaaeqabaqabeGadaaakeaadaWcaaqaaiabdsfaujabdcfaqbqaaiabdAeagjabdcfaqbaaaaa@3154@ ratio. In this paper, the following objective function was used:

f(Z)={0,TP<20%TP(Z)FP(Z),TP≥20%
 MathType@MTEF@5@5@+=feaafiart1ev1aaatCvAUfKttLearuWrP9MDH5MBPbIqV92AaeXatLxBI9gBaebbnrfifHhDYfgasaacH8akY=wiFfYdH8Gipec8Eeeu0xXdbba9frFj0=OqFfea0dXdd9vqai=hGuQ8kuc9pgc9s8qqaq=dirpe0xb9q8qiLsFr0=vr0=vr0dc8meaabaqaciaacaGaaeqabaqabeGadaaakeaacqWGMbGzcqGGOaakcqWGAbGwcqGGPaqkcqGH9aqpdaGabeqaauaabaqaciaaaeaacqaIWaamcqGGSaalaeaacqWGubavcqWGqbaucqGH8aapcqaIYaGmcqaIWaamcqGGLaqjaeaadaWcaaqaaiabdsfaujabdcfaqjabcIcaOiabdQfaAjabcMcaPaqaaiabdAeagjabdcfaqjabcIcaOiabdQfaAjabcMcaPaaacqGGSaalaeaacqWGubavcqWGqbaucqGHLjYScqaIYaGmcqaIWaamcqGGLaqjaaaacaGL7baaaaa@4D36@

where *Z *is a chromosome and *f *is the fitness function. This fitness function gives a higher fitness level to chromosomes that generate a higher TPFP
 MathType@MTEF@5@5@+=feaafiart1ev1aaatCvAUfKttLearuWrP9MDH5MBPbIqV92AaeXatLxBI9gBaebbnrfifHhDYfgasaacH8akY=wiFfYdH8Gipec8Eeeu0xXdbba9frFj0=OqFfea0dXdd9vqai=hGuQ8kuc9pgc9s8qqaq=dirpe0xb9q8qiLsFr0=vr0=vr0dc8meaabaqaciaacaGaaeqabaqabeGadaaakeaadaWcaaqaaiabdsfaujabdcfaqbqaaiabdAeagjabdcfaqbaaaaa@3154@ ratio. We also postulated that TP rates below 20% were too low for the successful operation of a self-paced BI system (since they correspond to detection of less than one IC out of every five IC states, which may lead to user frustration, even though the FP rates might be very low). Such chromosomes were considered "unfit" and were assigned a "0" fitness value.

Next, a lexicographic approach was applied for multi-objective optimization of the GA population [23]. Very briefly, in this approach, the objectives were ranked according to the priorities assigned to them prior to optimization. The objective with the highest priority was used first for comparing the members of the population. In our case, the average of TPFP
 MathType@MTEF@5@5@+=feaafiart1ev1aaatCvAUfKttLearuWrP9MDH5MBPbIqV92AaeXatLxBI9gBaebbnrfifHhDYfgasaacH8akY=wiFfYdH8Gipec8Eeeu0xXdbba9frFj0=OqFfea0dXdd9vqai=hGuQ8kuc9pgc9s8qqaq=dirpe0xb9q8qiLsFr0=vr0=vr0dc8meaabaqaciaacaGaaeqabaqabeGadaaakeaadaWcaaqaaiabdsfaujabdcfaqbqaaiabdAeagjabdcfaqbaaaaa@3154@ over the validation sets was first selected as the objective function with the highest priority. The chromosomes were then ranked in a single-objective fashion. Any ties were resolved by comparing the relevant chromosomes again with respect to objectives that were assigned lower priority. The other three objectives were chosen as (1) the average of FP rate over the validation sets, (2) the average of TP rate over the validation set, and (3) the number of features, resulting in four objectives per chromosome in the GA population. The 2^nd ^and 3^rd ^objectives were ordered such that for two chromosomes with the same TPFP
 MathType@MTEF@5@5@+=feaafiart1ev1aaatCvAUfKttLearuWrP9MDH5MBPbIqV92AaeXatLxBI9gBaebbnrfifHhDYfgasaacH8akY=wiFfYdH8Gipec8Eeeu0xXdbba9frFj0=OqFfea0dXdd9vqai=hGuQ8kuc9pgc9s8qqaq=dirpe0xb9q8qiLsFr0=vr0=vr0dc8meaabaqaciaacaGaaeqabaqabeGadaaakeaadaWcaaqaaiabdsfaujabdcfaqbqaaiabdAeagjabdcfaqbaaaaa@3154@ ratio, the one with the lower FP rate was considered to be the fit chromosome.

The remaining operators of the GA were tournament-based selection (tournament size = 3), uniform crossover and uniform mutation. The sizes of the initial population and the population in the next generations were chosen as 100 and 50, respectively. We used random initialization to initialize the GA. Elitism was used to keep the best performing chromosome of each population in the subsequent populations.

The number of evaluations was set to 2000. If the improvement in the TPFP
 MathType@MTEF@5@5@+=feaafiart1ev1aaatCvAUfKttLearuWrP9MDH5MBPbIqV92AaeXatLxBI9gBaebbnrfifHhDYfgasaacH8akY=wiFfYdH8Gipec8Eeeu0xXdbba9frFj0=OqFfea0dXdd9vqai=hGuQ8kuc9pgc9s8qqaq=dirpe0xb9q8qiLsFr0=vr0=vr0dc8meaabaqaciaacaGaaeqabaqabeGadaaakeaadaWcaaqaaiabdsfaujabdcfaqbqaaiabdAeagjabdcfaqbaaaaa@3154@ ratio of the best solution was found to be less than 1% for more than 10 consecutive generations, the algorithm was terminated. Because of the computational load, tuning the GA parameter values (such as the mutation and crossover rates) was not performed.

A support vector machine (SVM) that uses kernel-based learning was chosen to classify each chromosome in the GA population. In kernel-based learning, all of the beneficial properties of linear classification methods, such as simplicity, are maintained, however, the overall classification is nonlinear in the input space, since the feature and input spaces are nonlinearly related [35]. Another reason for selecting an SVM as a classifier is that SVMs not only minimize the empirical risk (training error), they also minimize the confidence error (test error) [36]. We used the LIBSVM software[37], which has also been used in other BI papers [38,39].

The evaluation process was as follows. For each subject, IC and NC epochs were randomized and divided into training, validation and test sets. The training set was used to train the classifier, and the validation set was used to select the best set of features. The configuration yielding the best results on the validation set in the multi-objective sense mentioned above was selected, and the performance of the system calculated on the test set was reported. We used a five-fold nested cross-validation for evaluating the performance of the system. For each outer cross-validation set, 20% of the data were used for testing and the rest were used for training and model selection (selection of optimal subset of features). In order to select the models, the datasets were further divided into five folds. For each fold, 80% of the data were used for training the classifier and 20% were used for model selection.

To deal with the problem of unbalanced training sets (there were at least 20 times more NC epochs than IC epochs), the size of the NC training feature set was reduced to be the same as the size of the training IC feature sets. This was done by randomly selecting epochs from the NC training set.

## Results

In this section, we present our offline analysis of the data of the four subjects described in the "Data Collection" Section. We performed a search on the classifier's parameters during the model selection. Our findings showed that a 5^th ^degree polynomial kernel function performed better than other kernel functions studied (linear, polynomial with a degree other than 5 (3, 4, 6 and 7) and RBF kernel).

Since a five-fold nested cross-validation was used for the performance evaluation, the results were averaged over five runs of the outer validation sets. The columns 1 to 5 of Table 2 show the subject identification number, the average TP rate on the test sets, the average FP rate on the test sets, the average TPFP
 MathType@MTEF@5@5@+=feaafiart1ev1aaatCvAUfKttLearuWrP9MDH5MBPbIqV92AaeXatLxBI9gBaebbnrfifHhDYfgasaacH8akY=wiFfYdH8Gipec8Eeeu0xXdbba9frFj0=OqFfea0dXdd9vqai=hGuQ8kuc9pgc9s8qqaq=dirpe0xb9q8qiLsFr0=vr0=vr0dc8meaabaqaciaacaGaaeqabaqabeGadaaakeaadaWcaaqaaiabdsfaujabdcfaqbqaaiabdAeagjabdcfaqbaaaaa@3154@ ratio and the average number of features selected by the hybrid feature selection process. The numbers in parentheses are the standard deviations. As Table 2 shows, low FP rates for three of the four subjects (subjects AB1, AB2 and AB4) were achieved for a relatively high TP rate. For subject AB3, the TP results on the test sets were low (although the FP rates remained less than 4%).

**Table 2:** Comparison of the average TP, average FP rates, average TPFP
 MathType@MTEF@5@5@+=feaafiart1ev1aaatCvAUfKttLearuWrP9MDH5MBPbIqV92AaeXatLxBI9gBaebbnrfifHhDYfgasaacH8akY=wiFfYdH8Gipec8Eeeu0xXdbba9frFj0=OqFfea0dXdd9vqai=hGuQ8kuc9pgc9s8qqaq=dirpe0xb9q8qiLsFr0=vr0=vr0dc8meaabaqaciaacaGaaeqabaqabeGadaaakeaadaWcaaqaaiabdsfaujabdcfaqbqaaiabdAeagjabdcfaqbaaaaa@3154@ and the average number of features.

**Subject ID**	**Test Set (Current Study)**			**Number of features (Current Study)**	**Test Set ([42])**			**Number of Features ([42])**
				
	**TP**	**FP**	TPFP MathType@MTEF@5@5@+=feaafiart1ev1aaatCvAUfKttLearuWrP9MDH5MBPbIqV92AaeXatLxBI9gBaebbnrfifHhDYfgasaacH8akY=wiFfYdH8Gipec8Eeeu0xXdbba9frFj0=OqFfea0dXdd9vqai=hGuQ8kuc9pgc9s8qqaq=dirpe0xb9q8qiLsFr0=vr0=vr0dc8meaabaqaciaacaGaaeqabaqabeGadaaakeaadaWcaaqaaiabdsfaujabdcfaqbqaaiabdAeagjabdcfaqbaaaaa@3154@		**TP**	**FP**	TPFP MathType@MTEF@5@5@+=feaafiart1ev1aaatCvAUfKttLearuWrP9MDH5MBPbIqV92AaeXatLxBI9gBaebbnrfifHhDYfgasaacH8akY=wiFfYdH8Gipec8Eeeu0xXdbba9frFj0=OqFfea0dXdd9vqai=hGuQ8kuc9pgc9s8qqaq=dirpe0xb9q8qiLsFr0=vr0=vr0dc8meaabaqaciaacaGaaeqabaqabeGadaaakeaadaWcaaqaaiabdsfaujabdcfaqbqaaiabdAeagjabdcfaqbaaaaa@3154@	
**AB1**	66.96 (4.79)	0.99 (0.39)	**67.64**	30.6 (1.14)	67.80 (1.4)	2.0	33.90	6
**AB2**	73.34 (2.63)	1.40 (0.42)	**52.39**	29.2 (3.27)	74.0 (1.7)	2.0	37.0	6
**AB3**	33.08 (14.03)	3.88 (1.04)	8.53	23.4 (2.41)	64.0 (1.3)	2.0	**32.0**	6
**AB4**	56.10 (4.90)	1.41 (0.75)	**39.79**	27.0 (2.83)	73.1 (1.8)	2.0	36.55	6

**Average**	57.37	1.92	29.88	27.55	69.73	2.0	34.86	6

**Figure 2:**
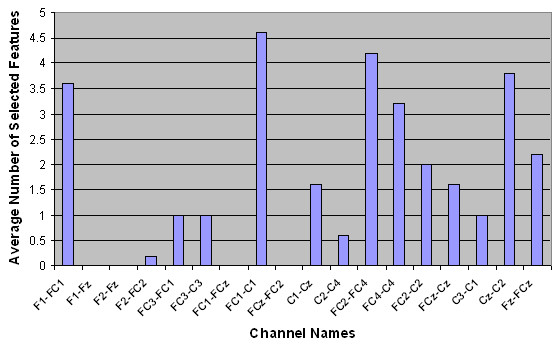
Spatial distribution of the average number of selected features for Subject AB1.

**Figure 3:**
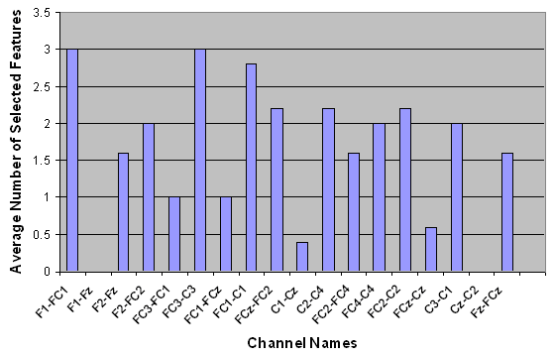
Spatial distribution of the average number of selected features for Subject AB2.

**Figure 4:**
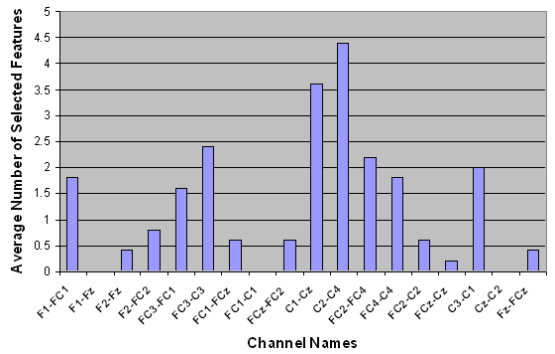
Spatial distribution of the average number of selected features for Subject AB3.

**Figure 5:**
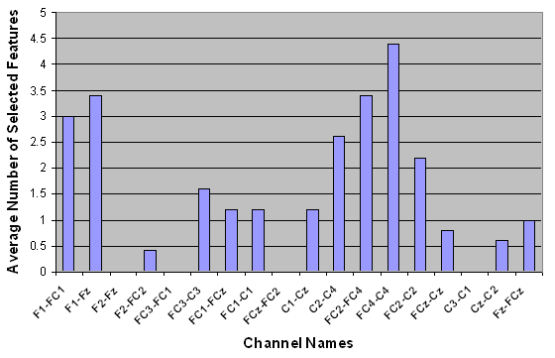
Spatial distribution of the average number of selected features for Subject AB4.

Next, the spatial distributions of the selected features were examined. The average number of selected features per channel is shown in Table 3. The numbers in parentheses show the standard deviation over five runs of outer cross-validation. Figures 2 to 5 show the number of selected features per channel for all subjects after applying the hybrid selection method (averaged over the number of cross-validation sets). The low standard deviation obtained for all cases shows the robustness of the proposed method over different runs of the algorithm.

**Table 3:** The average number of selected features per channel after applying the hybrid feature selection algorithm.

**Channel/Subject ID**	**AB1**	**AB2**	**AB3**	**AB4**
**F_1_-FC**_1_	3.6 (1.14)	3 (1.22)	1.8 (0.84)	3 (0.71)
**F_1_-F**_z_	0 (0)	0 (0)	0 (0)	3.4 (0.55)
**F_2_-F**_z_	0 (0)	1.6 (0.89)	0.4 (0.55)	0 (0)
**F_2_-FC**_2_	0.2 (0.45)	2 (0.71)	0.8 (0.84)	0.4 (0.55)
**FC_3_-FC**_1_	1 (0)	1 (0)	1.6 (0.89)	0 (0)
**FC_3_-C**_3_	1 (0.71)	3 (0)	2.4 (1.14)	1.6 (0.55)
**FC_1_-FC**_z_	0 (0)	1 (0)	0.6 (0.55)	1.2 (0.84)
**FC_1_-C**_1_	4.6 (0.55)	2.8 (0.45)	0 (0)	1.2 (0.45)
**FC_z_-FC**_2_	0 (0)	2.2 (0.45)	0.6 (0.55)	0 (0)
**C_1_-C**_z_	1.6 (0.55)	0.4 (0.55)	3.6 (1.14)	1.2 (0.45)
**C_2_-C**_4_	0.6 (0.55)	2.2 (0.45)	4.4 (0.89)	2.6 (0.89)
**FC_2_-FC**_4_	4.2 (0.45)	1.6 (0.89)	2.2 (1.10)	3.4 (1.14)
**FC_4_-C**_4_	3.2 (0.45)	2 (1)	1.8 (0.84)	4.4 (0.55)
**FC_2_-C**_2_	2 (0)	2.2 (0.45)	0.6 (0.55)	2.2 (0.45)
**FC_z_-C**_z_	1.6 (0.89)	0.6 (0.55)	0.2 (0.45)	0.8 (0.45)
**C_3_-C**_1_	1 (0.71)	2 (0)	2 (0)	0 (0)
**C_z_-C**_2_	3.8 (0.45)	0 (0)	0 (0)	0.6 (0.55)
**F_z_-FC**_z_	2.2 (1.30)	1.6 (0.55)	0.4 (0.55)	1 (0.71)

## Discussion and conclusions

Discrete wavelet transform (DWT) is a useful feature extraction tool since it explores the time as well as the frequency information of the signal. Although DWT has been employed to some degree of success in a number of synchronized BI systems, there remain some limitations in its application to self-paced BI systems (in terms of the large size of the feature space).

Brain interface systems that use DWT features have mostly employed only one or two channels (perhaps due to the large dimensionality of the feature space or to limitations imposed by the experimental protocol). To simultaneously explore the wavelet coefficients (features) of BIs with more channels (so as to explore the spatial information) and to avoid the problems associated with the resultant large feature space, a two-stage (hybrid) feature selection algorithm is proposed. The first stage uses mutual information (MI) to discard the least informative features. In the second stage, a genetic algorithm (GA) selects those remaining features that lead to better system performance in the sense of meeting multiple objectives.

In our study, the features selected per channel varied considerably from one subject to another, as shown in Figures 2 to 5. For example, for subject AB1, more features were selected from channels FC_1_-C_1_, F_1_-FC_1_, F_z_-FC_z_, FC_4_-C_4_, FC_2_-FC_4 _and C_z_-C_2_, while for subject AB4, more features were selected from channels FC_4_-C_4_, FC_2_-FC_4_, F_1_-F_z_, C_2_-C_4_, F_1_-FC_1_, and FC_2_-C_2_. These results support the hypothesis that proper channel selection for every subject is necessary to obtain superior performance.

Another finding from Figures 2 to 5 is that the relevant features for each subject were unique. These findings are in contrast to an earlier study done by our group that empirically determined six pairs of electrodes for all subjects (channels F_1_-FC_1_, F_2_-FC_2_, FC_1_-C_1_, FC_2_-C_2_, FC_z_-C_z_, and F_z_-FC_z_) [3]. Our findings in this regard are not surprising. The evidence from the literature supports the hypothesis that there is a significant amount of intersubject variability in terms of generating MRP patterns [40]. The literature also shows that the selected features are not necessarily located in the standard frequency bands or on specific scalp locations, and that the set of selected features differs from subject to subject [41]. These studies support the notion that a customized BI system should be designed for each subject.

Table 3 shows that for each subject, a number of bipolar channels were not selected by the feature selection process (such as channel F_1_-F_z _for subjects AB1, AB2 and AB3, and channel FC_3_-FC_1 _for subject AB4). These results indicate that these channels can be eliminated from the analysis in future studies. Moreover, Table 3 and Figures 2 to 5 show that the degree of contribution to the classification performance varies from one channel to another. These results indicate that a channel elimination methodology could be incorporated into the proposed method to further decrease the number of channels used for the operation of the system. This approach would rank the channels according to the number of selected features. It would then repeatedly eliminate the channel with the lowest contribution to fitness until the performance drops below a certain threshold (recursive elimination of channels). Systematic elimination of channels can lead to a faster setup of the system as well as decreased computational time. This could be part of future research works aimed at moving towards a more practical system.

It should be mentioned that it is difficult to directly compare the results of our study with other BI studies. This is because the number of subjects, the type of subject (whether or not subjects are able-bodied), the experimental protocols, the evaluation protocol and the neurological phenomenon differ from one study to another. In addition, the number of available EEG epochs, as well as the degree of training subjects receive before participating in a BI experiment, vary among studies.

We can, however, compare our current results with the latest design of a state-of-the-art self-paced BI system called the low frequency-asynchronous switch design (the LF-ASD) [42]. Both studies use the same subjects, the same experimental protocol, the same EEG data and the same evaluation protocol.

The LF-ASD (originally reported in [3] and later modified as reported in [42]) uses a feature extractor with a shape similar to a wavelet function, and extracts features from six bipolar EEG channels. The Karhunen-Loève Transform (KLT) is used to reduce the 6-dimensional feature space produced by the feature generator to a 2-dimensional space. A 1-NN classifier is used as the feature classifier. A moving average and a debounce algorithm are employed to improve the performance of the system by reducing the number of false activations. The parameter values of the system were estimated by an expert (for details, see [3,30,42]). The latest performance results of the LF-ASD [42], applied to the data of subjects AB1 to AB4 are presented in columns 6 to 9 of Table 2. As can be seen from the table, our proposed system has resulted in an increased TPFP
 MathType@MTEF@5@5@+=feaafiart1ev1aaatCvAUfKttLearuWrP9MDH5MBPbIqV92AaeXatLxBI9gBaebbnrfifHhDYfgasaacH8akY=wiFfYdH8Gipec8Eeeu0xXdbba9frFj0=OqFfea0dXdd9vqai=hGuQ8kuc9pgc9s8qqaq=dirpe0xb9q8qiLsFr0=vr0=vr0dc8meaabaqaciaacaGaaeqabaqabeGadaaakeaadaWcaaqaaiabdsfaujabdcfaqbqaaiabdAeagjabdcfaqbaaaaa@3154@ ratio for all subjects (with the exception of subject AB3). Specifically, the TPFP
 MathType@MTEF@5@5@+=feaafiart1ev1aaatCvAUfKttLearuWrP9MDH5MBPbIqV92AaeXatLxBI9gBaebbnrfifHhDYfgasaacH8akY=wiFfYdH8Gipec8Eeeu0xXdbba9frFj0=OqFfea0dXdd9vqai=hGuQ8kuc9pgc9s8qqaq=dirpe0xb9q8qiLsFr0=vr0=vr0dc8meaabaqaciaacaGaaeqabaqabeGadaaakeaadaWcaaqaaiabdsfaujabdcfaqbqaaiabdAeagjabdcfaqbaaaaa@3154@ ratio increased from 33.90 to 67.74 for subject AB1 (a relative improvement of 99.5%), from 37 to 52.39 for subject AB2 (a relative improvement of 41.6%), and from 36.55 to 39.79 for subject AB4 (a relative improvement of 8.9%). These results show that our proposed approach improved the performance of most subjects compared with the latest design of the LF-ASD. The degree of improvements in the TPFP
 MathType@MTEF@5@5@+=feaafiart1ev1aaatCvAUfKttLearuWrP9MDH5MBPbIqV92AaeXatLxBI9gBaebbnrfifHhDYfgasaacH8akY=wiFfYdH8Gipec8Eeeu0xXdbba9frFj0=OqFfea0dXdd9vqai=hGuQ8kuc9pgc9s8qqaq=dirpe0xb9q8qiLsFr0=vr0=vr0dc8meaabaqaciaacaGaaeqabaqabeGadaaakeaadaWcaaqaaiabdsfaujabdcfaqbqaaiabdAeagjabdcfaqbaaaaa@3154@ ratio, however, is not statistically significant (*p *> 0.05), so tests on the data of more subjects are needed to further substantiate this improvement. Note that the improved performance was achieved at the expense of using more features (please see columns 5 and 9 in Table 2).

The relatively poor results obtained for subject AB3 may be partly related to our choice of wavelet function. Note that the wavelet function chosen for this study was based on the similarities between the chosen wavelet function and a typical bipolar MRP ensemble average pattern. However, there is substantial inter-subject variability in the shape of MRPs, especially in single trials [42]. It is expected that by analyzing a more diverse family of wavelet functions, a different wavelet function might be chosen for each subject that would produce superior results.

**Figure 6:**
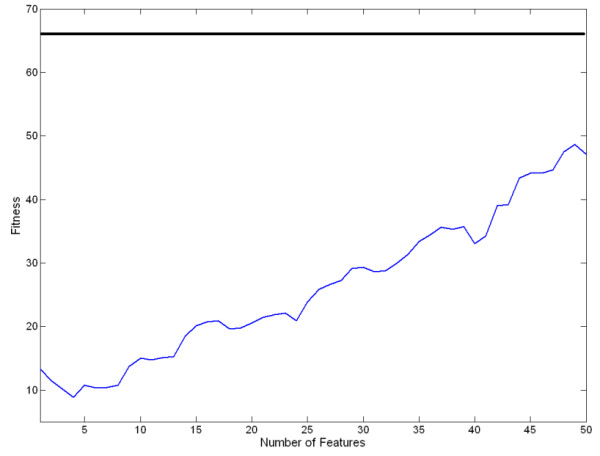
Comparison of the fitness of the best chromosome vs. other subset of features.

As mentioned in "Methods" Section, we designated the number of features chosen by the MI to be *L *= 50. Fewer features would have sped up the process of feature selection at the second stage, but might have resulted in a lower fitness value. To test this possibility, we compared the fitness of the best subset of features (see Table 2) with that of all features for subject AB1 (see Figure 6). In this figure, the black line shows the fitness of the best configuration (calculated from Table 2). The blue line shows the fitness of the classifier as a function of the number of top features. We began by training and testing the classifier using only the feature with the highest MI score, and then calculated the fitness. Then we added features one at a time (according to their MI scores) and trained and tested the classifier using the new set of features. This process was repeated until we reached *L *= 50. Although the fitness of the classifier increased as more features were added, it stayed well below the optimal value achieved by the GA. These results indicate that a lower *L *(especially when only limited top features are used for training the classifier) does not necessarily lead to better performance.

A useful area to explore is the automation of the classifier. Currently, the feature selection procedure is automated but the selection of other parameter valuea, such as those of the classifier, is carried out through cross-validation. Incorporating these parameters into the automation process would relieve the designer from the tiresome process of selecting the classifier's parameter values, while potentially yielding better classification results. Expanding the current results to continuous signals and ultimately online testing are also worthwhile topics for future work.

These results should be considered as preliminary results in the development of a self-paced brain interface system with a low FP rate. Our future work will also include testing of the proposed system on a larger pool of subjects to further investigate its usability.

## Abbreviations

Abbreviation Complete Name

AB Able-bodied

BI brain interface

CWT continuous wavelet transform

DWT discrete wavelet transform

EEG Electroencephalography

EMG Electromyography

EOG Electrooculography

ERP event-related potential

FN false negative

FP false positive

GA genetic algorithm

IC intentional control

MI mutual information

MRP movement-related potentials

NC no control

SCP slow cortical potentials

SVM support vector machine

TN true negative

TP true positive

WT wavelet transform

## Authors' contributions

MF proposed the method, carried out the data analysis and drafted the manuscript. GEB and RKW critically revised the proposed scheme, and were involved in drafting the manuscript. All authors read and approved the final manuscript.
